# Meta-analysis of exome-wide gene burden analysis of breast cancer susceptibility genes

**DOI:** 10.1038/s41523-025-00826-8

**Published:** 2025-10-03

**Authors:** Jennifer A. Collister, Karl Smith-Byrne, Joshua Atkins, Gillian Reeves, David J. Hunter

**Affiliations:** 1 Nuffield Department of Population Health, University of Oxford, Oxford, UK; 2 Cancer Epidemiology Unit, Nuffield Department of Population Health, University of Oxford, Oxford, UK; 3 Department of Epidemiology, Harvard TH Chan School of Public Health, Boston, MA USA

**Keywords:** Breast cancer, Cancer genetics

## Abstract

We provide an updated meta-analysis of rare variants identified by exome sequences and breast cancer risk in up to 74,127 cases and 748,181 controls, combining results from 12,695 cases from the Million Women Study with published summary statistics. Protein-truncating variants in established susceptibility genes *BRCA2*, *BRCA1*, *CHEK2*, *PALB2*, *ATM* and *MAP3K1* were associated with a risk of breast cancer, while *BARD1* and *ATRIP* met exome-wide significance for the first time.

## Main

Breast cancer is the leading cause of cancer death among women globally. Previous studies have identified several genes known to be associated with breast cancer risk, and a range of candidate genes^1–5^.

The Million Women Study (MWS) is a prospective cohort of women recruited through the UK National Health Service (NHS) breast screening programme from 1996 to 2001 and followed up through questionnaires and record linkage to routinely collected NHS data^6^. Whole-exome sequencing (WES) was generated in 12,695 breast cancer cases and 24,507 controls from blood samples donated between 2006 and 2012 (see Methods, Supplementary Data 1).

We derived masks for gene burden analyses based on a previous large meta-analysis, categorising deleterious variation into any protein-truncating variants (PTVs) and rare (MAF <0.001) missense variants, and further classifying predicted deleterious missense variants using Combined Annotation Dependent Depletion (CADD score >20) and Helix (Helix score >0.5)^1,7,8^.

**Fig. 1:**
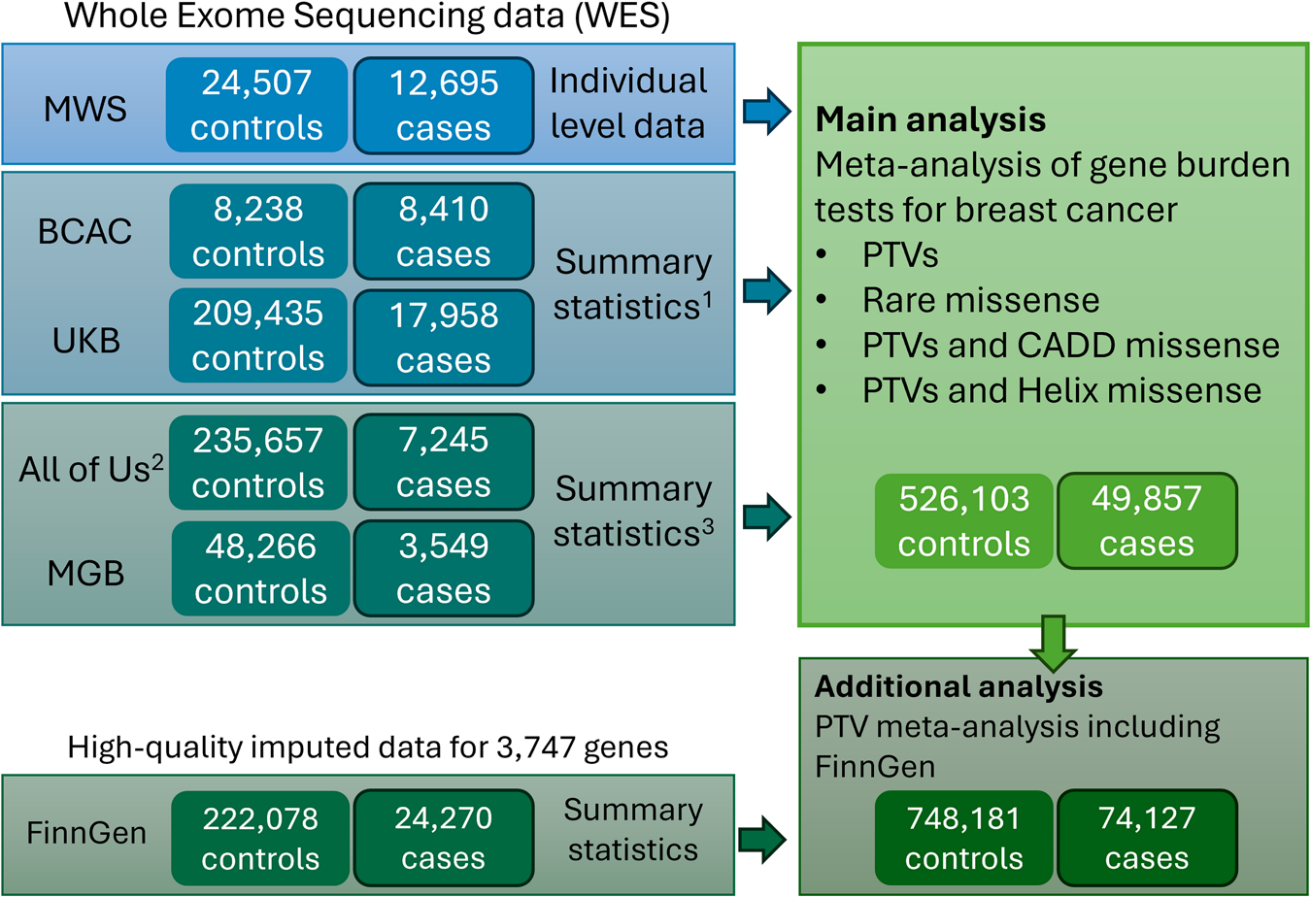
Overview of study design. MWS million women study, BCAC breast cancer association consortium, UKB UK Biobank, MGB Mass General Brigham Biobank. (1) The meta-analysis of BCAC and UKB used family history as a proxy for case status, incorporating data from men. (2) All of Us provides whole genome sequencing data (WGS), but for the purposes of this meta-analysis, the data were restricted to exonic variants. (3) The All of Us / MGB meta-analysis included both men and women in the cases and controls. The masks used in this meta-analysis were approximately paired with the masks used in the main analysis, see online Methods.

Results from these analyses were combined with results from two previous large WES gene burden meta-analyses: a largely European ancestry study including UK Biobank and 11 studies in the Breast Cancer Association Consortium (BCAC) (26,368 cases) and a study from the United States of America that included All of Us and the Mass General Brigham Biobank (10,794 cases)^1,9^. These combined studies now totalled to 49,857 cases and 526,103 controls (Fig. 1). In addition, we incorporated 24,270 cases from FinnGen, which provides gene-based association results for loss-of-function (LoF) variation in ~3.5k genes from high-quality imputed data^10^. In total, this analysis presents results based on up to 74,127 breast cancer cases and 748,181 controls.

In the MWS, we confirmed the previously reported gene burden associations of PTVs in *BRCA2*, *PALB2*, *BRCA1* and *CHEK2* and observed a suggestive association for ATM (Supplementary Data 2).

**Fig. 2:**
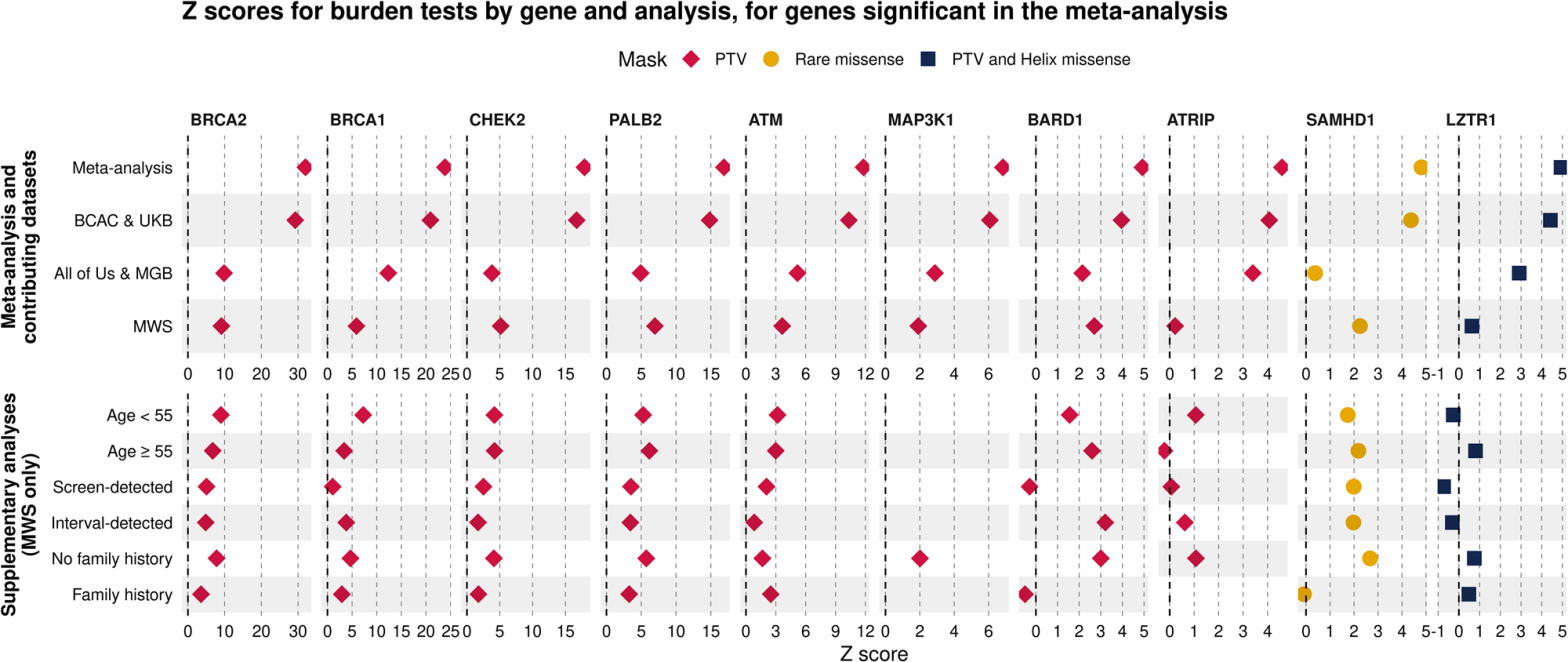
Forest plot of z-scores for burden tests by gene: in meta-analysis and contributing studies, and in supplementary analyses in the MWS cohort. Genes presented are those significant in either the PTV burden or any missense mask in the meta-analysis at Bonferroni-corrected exome-wide significance (Significance thresholds for PTV: *z* > 4.52, *P* < 6.20e-06, Rare missense: *z* > 4.68, *P* < 2.92e-06, PTV and CADD missense: *z* > 4.64, *P* < 3.54e-06, PTV and Helix missense: *z* > 4.60, *P* < 4.14e-06). Results shown are for the first mask significantly associated with each gene: Red diamonds are PTV, yellow circles are rare missense, navy squares are PTV and Helix missense. No association results are available for MAP3K1 in some supplementary analyses, as there were fewer than five PTV carriers across cases and controls within subgroups. PTV Protein-truncating variant, BCAC Breast Cancer Association Consortium, UKB UK Biobank, MGB Mass General Brigham, MWS Million Women Study.

In the meta-analysis of PTV burden, associations for six known breast genes met exome-wide significance (*P* < 6.20e-6): *BRCA2*, *BRCA1*, *CHEK2*, *PALB2*, *ATM*, *MAP3K1*, while candidate genes, *BARD1* and *ATRIP*, which were previously identified at a lower threshold now met exome-wide significance (Table 1 and Fig. 2)^1,3,11^. Results from missense variant masks also identified *CHEK2* and *SAMHD1* for rare missense variants, *CHEK2*, *BRCA2*, *PALB2*, *BRCA1* and *ATM* for combined PTV and CADD missense variants, and *BRCA2*, *BRCA1*, *CHEK2*, *PALB2*, *ATM*, *MAP3K1*, *LZTR1* and *BARD1* for PTV combined with Helix missense variants (Table 1 and Fig. 2). Suggestive genes associated at *P* < 0.001 for each mask are presented in Supplementary Data items 3–6.

**Table 1:** Association results by mask and gene for overall breast cancer, in MWS and in meta-analysis of MWS, BCAC, UKB, All of Us and MGB

Mask	Gene	MWS results				Meta-analysis	
		Carriers		Odds Ratio (95% CI)	*P*	Z-score	P
		Controls (*n* = 24,507)	Cases (*n* = 12,695)				
PTV	BRCA2	49 (0.20%)	117 (0.92%)	4.54 (3.22, 6.41)	6.47e-20	32.02	5.33e-225
	BRCA1	8 (0.03%)	33 (0.26%)	7.78 (3.55, 17.04)	3.73e-09	23.85	9.78e-126
	CHEK2	147 (0.60%)	141 (1.11%)	1.88 (1.48, 2.39)	2.86e-07	17.89	1.43e-71
	PALB2	26 (0.11%)	63 (0.50%)	4.75 (2.98, 7.57)	3.24e-12	16.89	5.64e-64
	ATM*	55 (0.22%)	56 (0.44%)	2.05 (1.40, 3.02)	2.77e-04	11.81	3.56e-32
	MAP3K1*	1 (<0.01%)	4 (0.03%)	6.25 (0.95, 41.03)	5.63e-02	6.84	8.07e-12
	BARD1*	16 (0.07%)	21 (0.17%)	2.47 (1.27, 4.83)	6.90e-03	4.93	8.28e-07
	ATRIP*	5 (0.02%)	3 (0.02%)	1.19 (0.26, 5.32)	8.23e-01	4.59	4.46e-06
	**Overall**	**306** (**1.25%)**	**430** (**3.39%)**	**2.78** (**2.38, 3.24)**	**6.72e-40**	–	–
Rare missense	CHEK2*	248 (1.01%)	171 (1.35%)	1.27 (1.04, 1.56)	1.95e-02	9.52	1.70e-21
	SAMHD1*	191 (0.78%)	129 (1.02%)	1.31 (1.04, 1.65)	2.46e-02	4.81	1.50e-06
	**Overall**	**437** (**1.78%)**	**297** (**2.34%)**	**1.29** (**1.11, 1.50)**	**1.30e-03**	–	–
PTV and CADD missense	CHEK2	345 (1.41%)	285 (2.24%)	1.58 (1.34, 1.86)	4.71e-08	18.90	1.13e-79
	BRCA2	444 (1.81%)	355 (2.80%)	1.58 (1.37, 1.83)	1.14e-09	16.84	1.34e-63
	PALB2	136 (0.55%)	128 (1.01%)	1.95 (1.52, 2.50)	1.91e-07	12.26	1.55e-34
	BRCA1*	232 (0.95%)	164 (1.29%)	1.40 (1.14, 1.73)	1.56e-03	11.73	8.52e-32
	ATM*	607 (2.48%)	379 (2.99%)	1.19 (1.04, 1.37)	9.89e-03	10.90	1.20e-27
	SAMHD1*	90 (0.37%)	60 (0.47%)	1.30 (0.92, 1.85)	1.36e-01	4.53	5.78e-06
	**Overall**	**1708** (**6.97)**	**1257** (**9.90%)**	**1.47** (**1.36, 1.59)**	**1.44e-21**	–	–
PTV and Helix missense	BRCA2	127 (0.52%)	163 (1.28%)	2.53 (1.99, 3.22)	3.52e-14	24.73	4.50e-135
	BRCA1	17 (0.07%)	44 (0.35%)	5.01 (2.81, 8.93)	4.28e-09	20.88	8.15e-07
	CHEK2	219 (0.89%)	211 (1.66%)	1.85 (1.52, 2.25)	1.17e-09	19.45	3.15e-84
	PALB2	28 (0.11%)	67 (0.53%)	4.78 (3.05, 7.50)	4.45e-13	14.81	1.18e-49
	ATM*	55 (0.22%)	56 (0.44%)	2.05 (1.40, 3.02)	2.77e-04	11.84	2.33e-32
	MAP3K1*	4 (0.02%)	14 (0.11%)	6.94 (2.27, 21.22)	1.22e-04	5.72	1.08e-08
	LZTR1*	125 (0.51%)	74 (0.58%)	1.10 (0.82, 1.49)	5.31e-01	4.93	8.25e-07
	BARD1*	22 (0.09%)	24 (0.19%)	2.24 (1.24, 4.07)	7.61e-03	4.79	1.67e-06
	**Overall**	**589** (**2.40%)**	**641** (**5.05%)**	**2.16** (**1.92, 2.43)**	**2.51e-37**	–	–

Genes presented are those significant in either the PTV burden or any missense mask in the meta-analysis at Bonferroni-corrected exome-wide significance (PTV: 6.20e-06, Rare missense: 2.92e-06, PTV and CADD missense: 3.54e-06, PTV and Helix missense: 4.14e-06). Carriers are women with at least one variant of the given category (mask) in the given gene. * Asterisk indicates genes that were not exome-wide significant within the MWS dataset.

*MWS* Million Women Study, *BCAC* Breast Cancer Association Consortium, *UKB* UK Biobank, *MGB* Mass General Brigham Biobank, *PTV* protein-truncating variants.

Bold text indicates overall values across presented genes for each mask. Overall carrier counts may be less than the sum of carrier counts per included gene, as individuals may be carriers of more than one gene.

In PTV meta-analyses including FinnGen^10^, the *BRCA2* association was attenuated, but *CHEK2* and *PALB2* were more strongly associated with breast cancer (Supplementary Data 7). The stronger association with *PALB2* is expected due to the c.1592delT founder mutation in the Finnish population, which drove the *PALB2* association in the FinnGen burden analysis.

Stratifying the MWS analysis by age at diagnosis of breast cancer, we observed that among carriers of *BRCA1* and *BRCA2* PTVs, the odds ratio (OR) for breast cancer aged <55 (vs controls) was more than double that of breast cancer aged ≥55. For PTVs in other known risk genes, including *PALB2 and CHEK2*, we observed a slightly stronger association with cancers diagnosed before age 55 (Supplementary Data items 8–11), consistent with previous work^3,12^.

We also investigated potential differences in genetic association with screen (*n* = 4508) vs interval (*n* = 1877) detected breast cancer in the MWS. Whilst the OR of PTV burden in *BRCA2* and *PALB2* for breast cancer remained broadly consistent between screening- and interval-detected cancers, PTV burden in both *RAD51C* and *BRCA1* had a markedly greater effect size and strength of association for interval-detected cancers (vs controls) than screen-detected cancers (Supplementary Data items 12–15). Previous work has shown that interval-detected cancers are more likely to be aggressive with a poorer prognosis than screen-detected cancers and also more likely to have PTVs in known breast cancer risk genes^13–15^.

In further analyses stratifying by first-degree family history of breast cancer, we observed, as expected, that PTVs in known risk genes such as *BRCA2*, *PALB2*, *BRCA1* and *CHEK2* were more prevalent among women with a family history of breast cancer (Supplementary Data items 16–19).

We acknowledge that there are several limitations to our study. The MWS and UK Biobank cohort will not capture many aggressive, early-onset cancers due to their recruitment ages (lower limit of 50 years and 40 years respectively), but other included datasets such as the BCAC and All of Us contribute some data on these women. Further, the population in this meta-analysis is predominantly European, which is a known problem in genetics research that may be addressed as newer cohorts like All of Us and Our Future Health mature and accrue sufficient cases.

In an analysis with almost twice as many cases as the last major breast cancer consortium meta-analysis^1^, we have strengthened the existing evidence for established and suspected risk genes. We confirmed that the relative risks for these gene variants decline sharply with age, and that the search for further novel genes of large effect is largely futile among women of European ancestry.

## Methods

### Million Women Study

The Million Women Study (MWS) is a population-based cohort recruited from women aged 50–64 who were invited for NHS breast cancer screening at 66 screening centres in England and Scotland from 1996 to 2001^6^. All study participants gave written informed consent to take part in the study, and ethical approval was provided by the Eastern Multi-Centre Research Ethics Committee.

Women received an initial questionnaire alongside their routine screening invitation, and follow-up questionnaires were sent to participants at 3–5 year intervals. Data linkage to hospital records, cancer and death registries enables continued follow-up of study participants.

During 2006–2012, participants in the MWS who had responded to the most recent postal resurvey (2nd questionnaire: 2006–2009, 3rd questionnaire: 2009–2012) were invited to provide a blood sample and participate in a genetic susceptibility study investigating breast cancer and heart disease. All women who self-reported a previous diagnosis of breast cancer were invited to participate, along with women who self-reported CVD in the second questionnaire and randomly selected controls.

#### Exome sample preparation, sequencing and QC

DNA was transferred to the Regeneron Genetics Center for whole-exome sequencing using an automated sample preparation approach as previously described^16–18^. DNA was enzymatically sheared to a mean fragment size of 200 bp to create DNA libraries, and a common Y-shaped adapter was ligated to all libraries. Samples were captured with Twist Bioscience’s Comprehensive Exome Panel, which targets 33 Mb of the human genome, including all protein-coding regions and UTRs. Captured libraries were sequenced on an Illumina sequencer using 75 bp paired-end reads and two index reads.

A total of 44,068 samples were received for processing. Regeneron were able to process 43,031 samples; the remainder failed due to low or no DNA being present. The average 20x coverage was 95.5%, and 99.6% of the samples were above 90%.

Of the 43,031 samples sequenced, 147 did not pass one or more of the Regeneron QC metrics and were subsequently excluded. Criteria for exclusion were as follows: disagreement between genetically determined and reported sex (*n* = 1); high rates of heterozygosity or contamination (VBID >5%) (*n* = 59); low sequence coverage (less than 80% of targeted bases achieving 20x coverage) (*n* = 20); genetically identified sample duplicates (*n* = 68 total samples). The remaining 42,884 samples were then used to compile a pVCF for downstream analysis, using the GLnexus joint genotyping tool.

#### Variant calling and quality control

Genotypes were called using Google’s DeepVariant genotyper, run with customised parameters tailored to Regeneron Genetics Centre exome sequence data.

#### Ancestry assignment

Genotyping-by-sequencing data, obtained from the Regeneron panel of genotyping by sequencing probes, was used to determine continental ancestry super-groups (African (AFR), Admixed American (AMR), East Asian (EAS), European (EUR) and South Asian (SAS)) by projecting each sample onto reference principal components calculated from the HapMap3 reference panel, as previously described^17^.

#### Analysis population

The breast cancer outcome was defined as a breast cancer diagnosis recorded in the cancer registry (ICD10: C50; ICD9: 174), irrespective of prior cancers. Both prevalent and incident cases were included.

To avoid sample overlap in the meta-analysis, we removed women who also participated in the UK Biobank study (*n* = 4173). We excluded women who had undergone a mastectomy (OPCS3: 382, 383, 384, 385, 3811, 3821; OPCS4: B271, B272, B273, B274, B275, B276, B278, B279) without a diagnosis of breast cancer (*n* = 536), those who had a diagnosis of carcinoma in situ (ICD10: D05; ICD9: 2330) without a breast cancer diagnosis (*n* = 970), and individuals missing both age at baseline and date of birth (*n* = 2).

This resulted in a dataset of 37,202 women, of whom 11,464 had a diagnosis of invasive breast cancer recorded in the cancer registry dated prior to receipt of the blood sample. A further 1231 individuals have a registered first breast cancer diagnosis after providing a blood sample.

Interval-detected breast cancers, defined as those diagnosed within 36 months after a scheduled screening attendance with a normal result, were categorised using the 'screeningstatusfull' variable from the Cancer Outcomes and Services Dataset (COSD). All cancers that were not labelled as either 'screen detected' or 'interval cancer' were considered to have unknown screening status.

#### Data preparation

In line with standard Regeneron QC, variants with locus missingness >10% or Hardy–Weinberg equilibrium (HWE) test *P* < 10^−15^ were excluded. The dataset was restricted to variants that fell within the target sequencing intervals. Variants were annotated using the Ensembl variant effect predictor (VEP) v110.1, and variant-specific consequences were determined as the most severe consequence of a MANE Select or canonical transcript in protein-coding genes.

The annotations were used to identify protein-truncating variants (PTVs) and rare missense variants. PTVs in the last exon of each gene and the last 50 bp of the penultimate exon were excluded in line with previous work^1^. Missense variants were further annotated with CADD and Helix scores to quantify their predicted deleteriousness^7,8^.

#### Burden tests

To interrogate rare variation, genetic variants were collapsed across genes in burden tests, as described in ref. ^17^. Regenie gene-based step 2 with the default ‘max’ mask option was used to test for an association between the burden of variation in each gene (the maximum number of alternate alleles across sites in a gene: 0 for individuals who are homozygous reference for all variants, 1 for individuals who are heterozygous for any variant, and 2 for individuals who are minor allele homozygotes for a variant), and breast cancer status.

For compatibility with previous work, four masks were used to capture potentially deleterious variation:

1. PTVs (Ensembl consequences: transcript_ablation, splice_acceptor_variant, splice_donor_variant, stop_gained, frameshift_variant).
2. Rare missense (Ensembl consequences: stop_lost, start_lost, transcript_amplification, inframe_insertion, missense_variant, protein_altering_variant, splice_region_variant; MAF <0.001 in analysis dataset and in 1000 genomes if present).
3. PTVs and rare missense with CADD score ≥20.
4. PTVs and rare missense with Helix score >0.5.

Gene burden tests were conducted for each mask in all genes in which there were at least five carriers (Regenie option minMAC = 5), adjusted for baseline age (except the age-stratified analysis) and the first ten principal components of genetic ancestry. The significance threshold was determined for each mask by Bonferroni correction for the number of genes included in the analysis.

### Other datasets

#### UKB

The UK Biobank (UKB) recruited over 500,000 individuals in England, Scotland and Wales from 2006 to 2010. All participants provided written informed consent to participate, and ethical approval was provided by the North West multi-centre Research Ethics Committee. After a detailed touchscreen questionnaire and verbal interview at baseline, follow-up information has been collected through repeat questionnaires and linkage to health records, and WES data has been released for over 450,000 participants^17^.

#### BCAC studies

The Breast Cancer Association Consortium is a research collaboration investigating the inherited risk of breast cancer, combining data from collaborating studies and research projects around the world. All studies were approved by local ethical review boards (Supplementary Data 20), and all subjects provided written informed consent. WES data from eight studies from the Breast Cancer Risk after Diagnostic Gene Sequencing (BRIDGES) dataset and three studies from the Personalised Risk assessment for prevention and early detection of breast cancer: integration and implementation (PERSPECTIVE) dataset were included, comprising a mixture of family history and carrier-based studies from Europe and South-East Asia^1^.

#### All of Us

The All of Us Research Programme is a cohort study in the United States that has been actively recruiting participants age 18+ since 2018, with a focus on enroling a diverse population including groups that have been historically underrepresented in research^19^. All enrolled participants provided informed consent; all data collection and analysis involving human participants were approved by the Institutional Review Board (IRB) of the All of Us Research Programme. Whole genome sequencing (WGS) has been performed on ~250,000 participants to date.

#### MGB

Mass General Brigham Biobank is an observational research study within Mass General Brigham, an integrated healthcare system in Boston, in the United States^20^. All adult patients provided informed consent to participate. A small number of children were enrolled with IRB-approved assent forms; upon reaching 18 years of age, all enrolled children had to provide consent or were removed from the study. The study received ethics approval from the IRB of Mass General Brigham. WES data is available for over 50,000 participants.

#### FinnGen

The FinnGen study is a large-scale genomics initiative that has analyzed over 500,000 Finnish biobank samples and correlated genetic variation with health data to understand disease mechanisms and predispositions^10^. The project is a collaboration between research organisations and biobanks within Finland and international industry partners. Participants in FinnGen provided informed consent for biobank research, based on the Finnish Biobank Act. Separate contributing research cohorts collected before the Finnish Biobank Act came into effect were collected based on study-specific consents and later transferred to the Finnish biobanks after approval by Fimea, the National Supervisory Authority for Welfare and Health. The study received ethics approval from the Co-ordinating Ethics Committee of the Hospital District of Helsinki and Uusimaa.

Individuals were genotyped using a custom Axiom FinnGen1 array (which included the PALB2 founder variant c.1592delT) and imputed to a population-specific reference panel of high-coverage WGS data. The cohort provides summary statistics from burden tests for loss-of-function (LoF) variants (Ensembl consequences: frameshift_variant, splice_donor_variant, stop_gained, splice_acceptor_variant; MAF <0.01; INFO >0.8) from their high-quality imputed data (INFO >0.6, MAF >0.0001). We downloaded summary results from Data Freeze 12, which included 3747 genes and had a breast cancer phenotype available for 246,348 women.

### Meta-analysis

For compatibility with the meta-analysis in ref. ^1^, the association tests for each gene were converted to z-scores ${z}_{j}$ for each study $j$, and the combined z-score ${z}_{M}$ was weighted by the PTV associations in the known risk gene *CHEK2*.

1 $${z}_{M}=\frac{\mathop{\sum }\limits_{j}{w}_{j}{z}_{j}}{\sqrt{\mathop{\sum }\limits_{j}{w}_{j}^{2}}}$$

where ${w}_{j}$ is the weight for study $j$, with weights $\left({w}_{1},{w}_{2},{w}_{3}\right)=\left(\frac{{z}_{1}}{{z}_{3}},\frac{{z}_{2}}{{z}_{3}},1\right)$.

The masks used in the meta-analysis of All of Us and MGB were slightly different, and were approximately paired with the masks from the BCAC/UKB meta-analysis as follows:

1. PTVs = ‘rare LoF’
2. Rare missense = ‘ultrarare missense 0.2’
3. PTVs and CADD missense = ‘rare LOF + missense 0.8’
4. PTVs and Helix missense = ‘rare LOF + missense 0.8’

Only genes present in all three studies were included in the meta-analysis.

When incorporating results from FinnGen in the meta-analysis, we paired their LoF mask with the PTV mask, as it was defined using almost identical Ensembl variant consequences. They had restricted to high-quality imputed variants (info score >0.8) and applied a maximum MAF of 0.01.

### Sensitivity analyses

In the meta-analysis of BCAC and UKB conducted by Wilcox et al, family history was used as a proxy for breast cancer case status^1^. As a sensitivity analysis, we used this methodology in the MWS, constructing a family history weighted case status variable

2 $$\mathrm{status}=\left\{\begin{array}{l}\,0.0,\,\mathrm{case}=0,\mathrm{family}\mathrm{history}=0\\ 0.5,\,\mathrm{case}=0,\mathrm{family}\mathrm{history}=1\\ 1.0,\,\mathrm{case}=1,\mathrm{family}\mathrm{history}=0\\ \,1.5,\,\mathrm{case}=1,\mathrm{family}\mathrm{hsitory}=1\,\end{array}\right.$$

and conducting a logistic regression analysis with genotype burden as the dependent variable and case status as the independent, adjusting for the first ten principal components. Genotype burden was defined as

3 $${G}_{i}=\left\{\begin{array}{l}0,\,\,\mathop{\sum }\limits_{j=1}^{p}{g}_{{ij}}=0\,\\ 1,\,\,\mathop{\sum }\limits_{j=1}^{p}{g}_{{ij}} > 0\end{array}\right.$$

where ${g}_{{ij}}=0,\,1,\,2$ is the number of alternate alleles observed for sample ${i}$ at variant $j$, and $p$ is the number of variants in the gene.

With this approach, we observed a slightly increased association for *CHEK2*, but very little impact on associations for the other genes (Supplementary Fig. 1).

Breast cancer risk varies by ethnicity, and the over-representation of European ancestry individuals in genetic research is a known issue. The MWS is a UK cohort, and when grouped into continental ancestry groups based on similarity to individuals from the 1000 Genomes Project using principal components analysis (PCA), only 223 women were not of European (EUR) ancestry. The BCAC/UKB meta-analysis was conducted among primarily European ancestry individuals^1^ and excluded UKB participants of non-European ancestry, but two of the contributing BCAC cohorts were Asian. The MGB is also predominantly European, but the All of Us cohort is much more diverse, with almost 50% of individuals having a genetically determined ancestry other than EUR^9^. We therefore conducted a sensitivity analysis restricted where possible to individuals of European ancestry by excluding women of non-European ancestry from the MWS and using the European-only summary statistics provided for the All of Us and MGB meta-analysis (Supplementary Data 21).

In our main analyses we excluded women with a diagnosis of carcinoma in situ and no breast cancer diagnosis, as these women are not representative of controls. Including these women as cases in the MWS makes little difference to the results (Supplementary Data 22).

In our age-stratified analysis, we compared women with breast cancer diagnosed before or after the age of 55 to all controls. Because of the recruitment age and long follow-up time in the MWS, all controls were older than 55 and so could not be age-stratified. To investigate depletion of susceptibles, we additionally computed the OR of breast cancer diagnosed before age 55 vs after age 55. The ORs from this case-case analysis were similar to the ratio of ORs from the case-control analyses

Although the MWS has some data on breast cancer subtypes (Supplementary Data 1), the numbers were unfortunately too small to allow robust statistical analysis.

## Supplementary information

Supplementary Information

Supplementary Data

## Data Availability

The design, methods, questionnaires and data access policy for the Million Women Study are available at https://www.ceu.ox.ac.uk/research/the-million-women-study. Summary statistics for the meta-analysis of BCAC data with UK Biobank were obtained from Wilcox et al., https://www.nature.com/articles/s41588-023-01466-z#data-availability. Summary statistics for the meta-analysis of All of Us with Mass General Brigham Biobank were obtained from Jurgens et al., https://www.nature.com/articles/s41588-024-01894-5#data-availability. Summary statistics from FinnGen are available on request from https://finngen.gitbook.io/documentation/data-download.

## Code availability

The code for this analysis is available at https://github.com/2cjenn/MWS_BurdenMeta.
