# Supplementary material for: Meta-analysis of exome-wide gene burden analysis of breast cancer susceptibility genes
Source: NPJ Breast Cancer. 2025 Oct 3;11:111. doi: 10.1038/s41523-025-00826-8 (PMC12494685; doi:10.1038/s41523-025-00826-8)
Supplement: Supplementary file 1 — Supplementary Information [file 41523_2025_826_MOESM1_ESM.pdf]

Supplementary Information

Contents

Supplementary Figure 1: Z score comparison between family history (FaH) as proxy for case status and conventional burden test in Regenie 1

Tables provided in Supplementary Data file: 2

Supplementary Figure 1: Z score comparison between family history (FaH) as proxy for case status and conventional burden test in Regenie

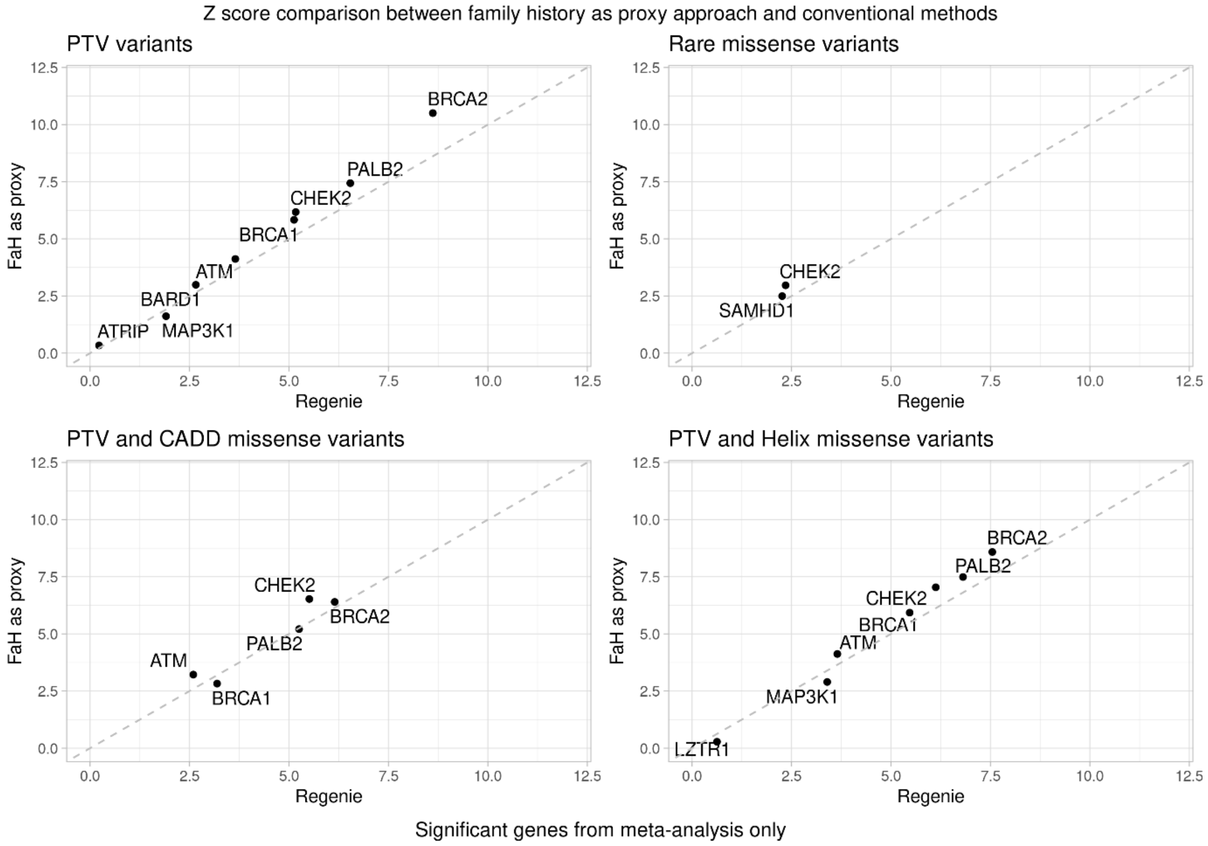

Tables provided in Supplementary Data file:

Supplementary Data 1: Summary of cases and controls in the MWS dataset.

Supplementary Data 2: Association results for overall breast cancer by mask in MWS (24,507 controls, 12,695 cases).

Supplementary Data 3: Association results for PTVs and overall breast cancer, in MWS and meta-analysis of MWS, BCAC, UKB, All of Us and MGB.

Supplementary Data 4: Association results for rare missense variants and overall breast cancer, in MWS and meta-analysis of MWS, BCAC, UKB, All of Us and MGB.

Supplementary Data 5: Association results for PTVs and CADD missense variants and overall breast cancer, in MWS and meta-analysis of MWS, BCAC, UKB, All of Us and MGB.

Supplementary Data 6: Association results for PTVs and Helix missense variants and overall breast cancer, in MWS and meta-analysis of MWS, BCAC, UKB, All of Us and MGB.

Supplementary Data 7: Association results for PTVs and overall breast cancer, in MWS and meta-analysis of MWS, BCAC, UKB, All of Us, MGB and FinnGen.

Supplementary Data 8: Association results in MWS for PTVs and breast cancer before and after age 55, compared to all controls.

Supplementary Data 9: Association results in MWS for rare missense variants and breast cancer before and after age 55, compared to all controls.

Supplementary Data 10: Association results in MWS for PTVs and CADD missense variants and breast cancer before and after age 55, compared to all controls.

Supplementary Data 11: Association results in MWS for PTVs and Helix missense variants and breast cancer before and after age 55, compared to all controls.

Supplementary Data 12: Association results in MWS for PTVs and screen- and interval-detected breast cancer, compared to all controls.

Supplementary Data 13: Association results in MWS for rare missense variants and screen- and interval-detected breast cancer, compared to all controls.

Supplementary Data 14: Association results in MWS for PTVs and CADD missense variants and screen- and interval-detected breast cancer, compared to all controls.

Supplementary Data 15: Association results in MWS for PTVs and Helix missense variants and screen- and interval-detected breast cancer, compared to all controls.

Supplementary Data 16: Association results in MWS for PTVs and breast cancer among women with or without family history, compared to controls.

Supplementary Data 17: Association results in MWS for rare missense variants and breast cancer among women with or without family history, compared to controls.

Supplementary Data 18: Association results in MWS for PTVs and CADD missense variants and breast cancer among women with or without family history, compared to controls.

Supplementary Data 19: Association results in MWS for PTVs and Helix missense variants and breast cancer among women with or without family history, compared to controls.

Supplementary Data 20: Ethics committees for the studies included in the meta-analysis.

Supplementary Figure 1: Z score comparison between family history (FaH) as proxy for case status and conventional burden test in Regenie

Supplementary Data 21: Association results for overall breast cancer by mask, in overall meta-analysis and meta-analysis of primarily European ancestry women from MWS, BCAC, UKB, All of Us and MGB.

Supplementary Data 22: Association results for overall breast cancer by mask in MWS, including carcinoma in situ among cases (24,507 controls, 13,665 cases).
